# Spectrum and the* In Vitro* Antifungal Susceptibility Pattern of Yeast Isolates in Ethiopian HIV Patients with Oropharyngeal Candidiasis

**DOI:** 10.1155/2016/3037817

**Published:** 2016-01-05

**Authors:** Birhan Moges, Adane Bitew, Aster Shewaamare

**Affiliations:** ^1^Department of Microbiology Quality Control, Ethiopian Food, Medicine and Healthcare Administration and Control Authority, Addis Ababa, Ethiopia; ^2^Department of Medical Laboratory Sciences, College of Health Sciences Addis Ababa University, Addis Ababa, Ethiopia; ^3^Department of Disease Prevention and Control, Zewditu Memorial Hospital, Addis Ababa, Ethiopia

## Abstract

*Background.* In Ethiopia, little is known regarding the distribution and the* in vitro* antifungal susceptibility profile of yeasts.* Objective.* This study was undertaken to determine the spectrum and the* in vitro* antifungal susceptibility pattern of yeasts isolated from HIV infected patients with OPC.* Method.* Oral pharyngeal swabs taken from oral lesions of study subjects were inoculated onto Sabouraud Dextrose Agar. Yeasts were identified by employing conventional test procedures and the susceptibility of yeasts to antifungal agents was evaluated by disk diffusion assay method.* Result.* One hundred and fifty-five yeast isolates were recovered of which 91 isolates were from patients that were not under HAART and 64 were from patients that were under HAART.* C. albicans *was the most frequently isolated species followed by* C. glabrata, C. tropicalis, C. krusei, C. kefyr, Cryptococcus laurentii, and Rhodotorula *species. Irrespective of yeasts isolated and identified, 5.8%, 5.8%, 12.3%, 8.4%, 0.6%, and 1.3% of the isolates were resistant to amphotericin B, clotrimazole, fluconazole, ketoconazole, miconazole, and nystatin, respectively.* Conclusion.* Yeast colonization rate of 69.2% and 31% resistance to six antifungal agents was documented. These highlight the need for nationwide study on the epidemiology of OPC and resistance to antifungal drugs.

## 1. Introduction

Human oral cavity is inhabited by many different kinds of microorganisms whose composition, metabolic activity, and pathogenicity are affected by external and internal factors [1, 2]. Among fungi, yeasts belonging to the genus* Candida* are considered to comprise the majority of fungal species present in the oral cavity.* Candida* species are the major causes of mucocutaneous infections that are usually classified as oropharyngeal, esophageal, and vulvovaginal candidiasis. Oropharyngeal candidiasis (OPC) is the commonest mucocutaneous candidiasis amongst HIV-positive patients worldwide [1, 3, 4], occurring in more than 95% of AIDS patients [1, 5]. High viral load, low CD4+ T lymphocyte count, and disease progression have been incriminated as the greatest risk factors for the development of OPC [4–6]. Although OPC infection is caused by many different species of yeast with the genus* Candida*,* C. albicans* remains the most predominant reported species globally [7].

Opportunistic fungal infections resistant to antifungal agents have been increasingly documented and there are concerns that their frequency will likely continue to increase. Müller et al. [8] and Pelletier et al. [9] demonstrated that emergence of resistance to antifungal agents in HIV/AIDS patients with OPC is common. Increased use of antifungal agents to treat fungal infections that occur in immune-compromised patients has been incriminated as a major factor for emergence of drug resistant yeast isolates, failure to respond to antifungal treatment with appropriate doses for a standard duration of time [10], and noticeable shift towards non-*albicans Candida* species with relative resistance to azole antifungal agents and associated refractory and recurrent infections [11, 12].

Although the introduction of Highly Active Antiretroviral Therapy (HAART) has dramatically reduced the incidence of opportunistic infections [13–15] in HIV-positive individuals who have received antifungal drugs, OPC with a shift in the spectrum of* Candida* species remains the most frequent HIV associated oral lesion [16] in developing countries including Ethiopia. Emergence of non-*albicans Candida* species as etiologic agents of mucocutaneous candidiasis and development of drug resistance by* C. albicans* to the currently available antifungal principles have initiated further studies in isolation and identification of the causative fungal species and determining of their antifungal profile. In Ethiopia, fungal culture and* in vitro* drug susceptibility pattern of yeast are not performed routinely. Therefore, little is known regarding the distribution and the* in vitro* antifungal susceptibility profile of yeasts isolated from HIV infected patients with OPC. To this end, determining species distribution of yeast isolates from Ethiopian HIV infected patients with and without HAART and evaluating their drug susceptibility profile to antifungal drugs is one of the highest priorities.

## 2. Materials and Methods

### 2.1. Study Area, Period, and Study Subjects

The present study was a single institutional cross-sectional study carried out in Zewditu Memorial Hospital, Addis Ababa, Ethiopia, over ten moths (September 2013 to June 2014). The hospital is one of the dedicated centers in the city for the management of HIV/AIDS patients and runs both outpatient and in-patient services. The HIV clinic of the hospital consists of an integrated counseling and testing center in addition to CD4+ T cells monitoring laboratory and ART pharmacy. Two hundred and twenty-four consecutive HIV infected patients with OPC, 112 under HAART and 112 not under HAART, were recruited when they were referred to the hospital for voluntary HIV counseling and testing (VCT) center and/or for outpatient departments (OPD) and/or referred from other health institution. Inclusion criteria were a previous positive diagnosis of HIV, will to participate in the study, a presumptive diagnosis of OPC following an appropriate complaint made at the clinic visit, and no history of antifungal therapy within two weeks prior to the attendance.

### 2.2. Specimen Collection and Transportation

Upon admission to the study, oral examination of each patient was performed and then oropharyngeal swabs were taken from oral lesions, oral cavity mucosa, and dorsal surface of the tongue and the floor of the mouth of each study subject with sterile rayon tipped applicator stick swabs. All samples were then inoculated into brain-heart infusion broth (Oxoid, Basingstoke, UK) supplemented with chloramphenicol (50 mg/L). Tubes of inoculated brain heart infusion were then transported to the Microbiology Laboratory of the Department of Medical Laboratory Science, College of Health Sciences, and Addis Ababa University, and incubated at 35–37°C for 18 to 24 hours.

### 2.3. Inoculation and Incubation

The brain-heart infusion broth was then subcultured onto Sabouraud Dextrose Agar (SDA) supplemented with chloramphenicol (Oxoid, Basingstoke, UK) and incubated aerobically for 48 hours. Plates with no growth after 48 hours were reincubated for a further 24 hours.

### 2.4. Identification

Yeasts were identified by employing conventional biochemical and assimilation test procedures [17], using CHROMagar* Candida* culture medium (Becton Dickinson) as per the instruction of the manufacture, germ-tube formation in human serum, and production of blastoconidia, pseudohyphae, and chlamydospore on cornmeal agar (Oxoid, Basingstoke, UK).

### 2.5. Determination of the Antifungal Profile of Yeast Isolates

The* in vitro* antifungal susceptibility pattern of yeast isolates to six antifungal agents, fluconazole, ketoconazole, miconazole, amphotericin B, clotrimazole, and nystatin (USP, Rockville, USA) was determined by agar diffusion procedure according to antifungal disk potency of Rosco Diagnostica Company (Neosensitabs, Denmark) [18]. All antifungal drugs were generously provided as standard powder by Ethiopian Food Medicine and Healthcare Administration and Control Authority (EFMHACA).

### 2.6. Impregnation of Antibiotic Assay Disk

The stock solutions were prepared by dissolving standard powders of each drug in their specific solvents (DMSO, water, and ethanol), after which they were impregnated into 6 mm sterile blank antibiotic assay disks (Sigma Aldrich, Germany) at the following potencies: clotrimazole 10 *μ*g/disk, fluconazole 25 *μ*g/disk, ketoconazole 10 *μ*g/disk, miconazole 10 *μ*g/disk, amphotericin B 10 *μ*g/disk, and nystatin 50 *μ*g/disk according to antifungal disk potency of Rosco Diagnostica Company (Neosensitabs, Denmark).

### 2.7. Inoculum Preparation

Yeast inocula were prepared by suspending five colonies of each isolate from 24-hour old pure culture in sterile saline and suspensions were then adjusted to the turbidity of a 0.5 McFarland standard to obtain 1 × 10^6^ to 5 × 10^6^ cells per mL. Yeast suspensions were then streaked onto Muller Hinton agar (Oxoid, Basingstoke, UK) supplemented with 2% glucose and 0.5 *μ*g/mL methylene blue (GMB). Dried antibiotic assay disks impregnated with antifungal agents as described above were placed on Muller Hinton agar seeded with yeast isolates after the plates were dried at ambient temperature for 15 minutes. Diameter zone of inhibition was measured by a metal caliper after 24 and 48 h of incubation at 35°C. Filter paper disks soaked in respective solvents without antifungal agent were placed on culture plate to serve as control. Standard strains of* C. albicans* ATCC 10231 were used for quality control. Criteria of susceptibility and resistance of antifungal agents were determined according to zone diameter interpretation for fungi of Rosco Diagnostica Company (Neosensitabs, Denmark) as shown in Table 1.

### 2.8. Statistical Analysis

Data were captured in MS Excel and analyzed using SPSS version 20 (IBM, Chicago, USA). Categorical variables were summarized by proportions and percentages. Comparisons of proportion between groups were carried out by chi-square test and the significant level set at *P* < 0.05.

### 2.9. Ethical Clearance

All ethical considerations and obligations were duly addressed and the study was conducted after the approval of the Department Research and Ethical Review Committee (DRERC) of the Department of Medical Laboratory Sciences, College of Health Sciences, and Addis Ababa University and the Addis Ababa City Health Bureau. Informed written consent was obtained from participants before data collection. The respondent was given the right to refuse to take part in the study and to withdraw at any time during the study period. All the information obtained from the study subjects were coded to remain confidentially. When the participants were found to be positive for fungal pathogen, they were informed by the hospital clinician and received proper treatment. Assent form was completed and signed by family member and/or adult guardian for participants under the age of 18 years.

## 3. Result

### 3.1. Patient Characteristics

In the present study, a total of 224 clinical samples were collected from HIV-sero-positive individuals with OPC of which 155 (69.2%) were from female patients and 69 (30.8%) were from male patients. The ages of study subjects ranged from 3 to 78 years with a median age of 34.5 years. Among the study participants, 112 (50%) were under HAART among which 76 (49%) were females and 36 (52.2%) were males. Details regarding demographic patient characteristics are given in Table 2.

### 3.2. Distribution of Yeast Isolates

Out of a total of 224 study subjects, 155 yeast isolates were recovered giving a prevalence of 69.2% colonization rate, of which 105 (67.7%) were from female patients and 50 (33.3%) were from male patients (Table 3). Differences in the isolation rate between gender were not statistically significant [(OR = 0.750, 95% CI, 0.414–1.36) (*P* = 0.342)].

Of one hundred and fifty-five (155) yeast isolates recovered from HIV patients with OPC, ninety-one (91) isolates were from patients that were not under HAART and 64 isolates were from patients that were under HAART (Table 4). Difference in yeast isolation rate between patients under HAART and those that were not under HAART was statistically significant [(OR = 2.433, 95% CI = 1.596–4.240) (*P* = 0.002)]. Species distribution as presented in Table 4 showed that* C. albicans* was the most frequently isolated species accounting 106 (68.4%) of the total yeast isolates followed by* C. glabrata*, 24 (15.5%),* C. tropicalis*, 17 (11.0%),* C. krusei*, 4 (2.6%), and* C. kefyr*, 2 (1.3%).* C. laurentii*, 1 (0.65%), and* Rhodotorula* species, 1 (0.65%), were the only isolated non-*Candida* species from these patients. Sixteen (16) patients had mixed infections and* C. albicans* appeared in all mixed infections.* C. albicans* appeared in seven patients with* C. glabrata* and in five patients with* C. tropicalis, C. krusei, C. kefyr, and C. laurentii, *and* Rhodotorula* species appeared once with* C. albicans*.

### 3.3. *In Vitro* Susceptibility of Isolates

The susceptibility of yeast isolates to the six antifungal drugs is summarized in Table 5. As can be shown from the table, 83.9% (130/155) of the total yeast isolates were susceptible, 10.3% (16/155) were intermediate, and 5.8% (9/155) were resistant to amphotericin B. One hundred and forty-eight (95.5%), 16 (10.3%), and 9 (5.8%) of yeast isolates were susceptible, intermediate, and resistant to clotrimazole, respectively. Yeast isolates were more resistant to fluconazole in which 19 (12.3) were resistant, while 123 (79.4%) and 13 (8.4%) were susceptible and intermediate, respectively. One hundred and thirty-four (86.5%), 8 (5.2%), and 13 (8.4%) of yeast isolates were susceptible, intermediate, and resistant to ketoconazole. More intermediate and least resistant yeasts were recorded against miconazole 21 (13.5%) and 1 (0.6%) was being intermediate and resistant against the drug, S: susceptible; I: intermediate; R: resistant; Am: amphotericin B; Clo: clotrimazole; Flu: fluconazole; Keto: ketoconazole; Mic: miconazole; NY: nystatin, while 133 (85.8%) were being susceptible to the drug. More yeast isolates were susceptible to nystatin in which 150 (96.8) were being susceptible, while 3 (1.9%) and 2 (1.3%) were intermediate and resistant to the drug, respectively. Nystatin was the most effective drug against* C. tropicalis* and* C. krusei* with no resistant strains of both species to the drug being observed. Nystatin also exhibited the greatest activity against* C. albicans* with no resistant strain found; overall, only one out of 106* C. albicans* isolates (0.9%) was found out to be intermediate, while 105 (99.1%) were susceptible to this agent. Nystatin was also relatively active drug against* C. glabrata* to which 22 (91.6%) out of 24 isolates were susceptible to the drug. Among the three major isolates of the present study,* C. tropicalis* was less sensitive to amphotericin B (53.0%), while the sensitivity of* C. albicans and C. glabrata* to the drug was 92.4% and 87.5%, respectively. Fluconazole was relatively the least effective drug against* C. albicans, C. glabrata,* and* C. tropicalis* which were the major isolates of this study. The sensitivity of* C. albicans, C. glabrata*, and* C. tropicalis* to the drug was 89.6%, 79.2%, and 58.8%, respectively. None of the isolates of* C. kefyr and C. krusei were* susceptible to ketoconazole, but the sensitivity of* C. albicans, C. glabrata*, and* C. tropicalis* to the drug was 89.6%, 83.3, and 58.8%, respectively. The sensitivity of* C. albicans, C. glabrata*, and* C. tropicalis* to clotrimazole was 99.1%, 87.5%, and 882%, respectively. Among the azole antifungal agents, more intermediate strains were produced against miconazole in which 100% of* C. krusei* and* C. kefir*, 35.5% of* C. tropicalis*, 20.8%* of C. glabrata,* and 3.8%* of C. albicans* were intermediate to the drug.

## 4. Discussion

Little is known about the profile and the* in vitro* antifungal susceptibility pattern of oral yeasts in Ethiopian HIV infected patients with OPC. Our finding of* C. albicans* as the predominant species was consistent with similar earlier [6, 19–25] and recent [26, 27] studies conducted internationally. Our finding of* C. albicans* as the predominant species was also consistent with similar study conducted locally in Gondar, southwest Ethiopia [28], but there were some striking differences in the species spectrum and percentage of yeast isolates. For instance, the culture positivity rate obtained in our study (69.2%) was comparatively lower than the culture positivity rate (82.3%) reported in the study carried out in Gondar. Differences in the recovery rate of different species of yeasts were also observed between these two studies. The recovery rate for* C. albicans* in our study was 106 (68.4%), 24 (15.5%) for* C. glabrata*, 17 (11%) for* C. tropicalis*, 4 (2.6%) for* C*.* krusei*, and 2 (1.3%) for* C. kefyr*. In the study conducted in Gondar, the recovery rate of different species of yeasts was higher, 139 (78.5) for* C. albicans*, 40 (22.5%) for* C. glabrata*, 17 (14.1%) for* C. tropicalis*, and 10 (5.6) for* C. krusei*, respectively. Similarly, their study apparently could not isolate* C. kefyr, C. laurentii*, and* Rhodotorula* species that made 2.6% of yeast isolates in our study.* C. kefyr* and other yeasts have been isolated as one of the oral yeasts by other works [6, 19]. Disparity in species spectrum and percentage of isolation rates of different yeasts between the two studies could be resulting from differences in study subjects enrolled in the two studies since half of our study subjects were under HAART. A marked decrease in the incidence of oral candidiasis in patients receiving HAART was reported by many earlier studies [14, 15, 29].

Although numerous studies on the prevalence of different* Candida* species have led to the general consensus that* C. albicans* is the most commonly isolated species in the oral cavity of patients infected with HIV, there has been a growing trend of recovery of non-*albicans Candida* species. This is evident by the present study in which the isolation rate of non-*albicans Candida* species was 30.8%. Comparatively, less recovery rates of non-*albicans Candida* species of 15% in Tanzania [6], 19% in Ethiopia [28], 22% in Texas [30], and 16.5% in Mexico [19] have been reported. Similarly, comparatively higher recovery rates of 55% and 50% of non-*albicans Candida* species have been reported in similar studies conducted in Nigeria [27] and Brazil [20], respectively.

A total of 155 isolates were obtained from 224 specimens during the 10-month period. Of clinical specimens collected 7.1% (16/224) yielded more than one yeast (mixed culture) species in which* C. albicans* was isolated from all mixed cultures. Four (1.5%) and forty (18.6%) mixed infections were reported by similar studies conducted in Tanzania [8] and Ethiopia [28], respectively. Fifteen percent (15%) and 29% mixed infections of* Candida* species among HIV patients were also reported by Baumgartner et al. [31] and Schoofs et al. [32], respectively. Our finding regarding the prevalence of mixed culture may reflect the change from single to multiple* Candida* species isolates, which have been responsible for epidemiological shift in OPC. The most predominant species isolated together with* C. albicans* in the present study was* C. glabrata* (43.8%). The significance of this finding is that* C. glabrata* may replace* C. albicans* under selective pressure of fluconazole, resulting in infections refractory to the current fluconazole based treatment in Ethiopia. Like other African countries, the present guideline of the Ethiopian Ministry of Health (MOH) for the management of candidiasis includes fluconazole as a first choice drug and ketoconazole, miconazole ointment as alternative antifungal agents [33, 34].

In this study, the occurrence of OPC was higher in the non-HAART group than in the HAART group. Of one hundred and fifty-five (155) yeast isolates recovered from HIV patients with OPC, ninety-one (91) isolates were from patients that were not under HAART and 64 isolates were from patients that were under HAART. This finding was consistent with previous reports from other countries. Ceballos-Salobrena et al. [14], in their study of 154 patients who were on HAART for a minimum of 6 months, reported a 30% reduction in the prevalence of OPC. Ramirez-Amador et al. [15], in their 12-year prospective study in Mexico, observed a decrease in the prevalence of OPC by half during the course of their defined study periods.

In the present study, the number of female patient participants (155, 69.2%) was greater than male patient participants (69, 30.8%). The significant difference in the number of female-male study participants could be due to the fact that female patients are more exposed to HIV infection than male patients.

Opportunistic fungal infections resistant to antifungal agents have been increasingly documented and their frequency will likely continue to increase [35]. This phenomenon appears largely due to the extensive use of antifungal agents to treat fungal infections that typically occur in severely immune-compromised and/or critically ill patients.* Candida* spp.,* Cryptococcus neoformans*, and* Aspergillus* spp. are among the leading fungi responsible for these invasive infections [36]. While antifungal resistance has been described with each of these fungi, resistance among* Candida* constitutes by far the most significant problem [37]. This was evident by the present study in which 31% (48/155) yeast isolates were found out to be resistant to one or more antifungal drugs tested. This result was comparable with earlier study conducted in Ethiopia [28] but relatively higher than similar studies from Africa [6, 21] and the rest of the world [38]. Of the 48 yeast isolates which were resistant to one or more drugs more than 64.6% (31/48) of the isolates were non-*albicans Candida* and this may reflect the intrinsic less susceptibility of some of the non-*albicans Candida* species to some antifungal agents such as fluconazole [39] and/or the reflection of the inappropriate use of antifungal in study area. Amphotericin B is traditionally used in topical formats, although it may be administrated systemically for the treatment of systemic infections in hospitalized patients. Our result on* in vitro* susceptibility of* Candida* species to polyene antifungal agents (amphotericin B and nystatin) was consistent with that reported previously [20] and showed that* Candida* isolates recovered in the study were highly sensitive to these polyene antifungal agents. Out of 155 yeast isolates, only 2 (1.3%) and 9 (5.8%) isolates were resistant to nystatin and amphotericin B, respectively. These results would support the effectiveness of topical amphotericin B and nystatin therapy for superficial candidosis. Recurrent OPC and repeatedly and/or prolonged exposure of patients to antifungal therapy induce reduced susceptibility of yeasts to azole antifungal agents [8, 9]. Selective pressure caused by antifungal agents and azole cumulative doses due to exposure to several courses of short- or long-term suppressive therapies in patients with OPC has been incriminated as a cause of induction of reduced susceptibility of yeasts to azole antifungal agents [9, 10]. Similarly, the risk of developing OPC with reduced susceptibility to azoles has been associated with greater duration of HIV infection and severe immunosuppression [10]. Isolation of yeasts more resistant to azole antifungal agents in the present study could be explained based on the above risk factors. Regardless of yeast identity, 19 (12.3%), 13 (8.4%), and 4 (2.6%) isolates were resistant to fluconazole, ketoconazole, and clotrimazole, respectively. Fluconazole was also the least active drug against* C. albicans* which is the most frequently isolated yeast. Though fluconazole has been widely used for the treatment of mucosal candidiasis because of its low toxicity and ease of administration local [28], many international studies have reported fluconazole resistance in* Candida* strains isolated from HIV infected patients with OPC [40–42]. Although we have no immediate reasons for a higher resistance of yeast isolates to fluconazole in the present study as indicated above, the accessibility of the drug being free of charge with the scale-up of HAART since 2005 in the country and the frequent usage of the drugs as the drug of choice in the settings could be responsible. Magaldi et al. [43] reported that 9.8%* C. albicans* isolated from patients with no previous treatment were resistant to fluconazole which increased to 44.7% after treatment with this drug. This data suggest that the continued use of fluconazole may lead to clinical treatment failure which is significantly correlated with reduced susceptibility to fluconazole and other azoles. In general, our study has shown that polyene antifungal (amphotericin B and nystatin) was more active than azole antifungal agents. Therefore, it is advisable to use these drugs as a second-line therapy for the mucosal candidiasis which is resistant to fluconazole or for empiric therapy as reported in other studies [12]. Only one isolate out of the 155 yeast isolates was found out to be resistant to miconazole, but 21 (13.5%) of the isolates were found out to be intermediate to the agent and why more isolates were found out to be intermediate to the agent is not clear and thus further study is recommended.

## 5. Conclusion

This study demonstrated that although* C. albicans* is the most frequently isolated species from HIV patients that are under HAART and not under HAART, high prevalence of mixed culture in this study may reflect the change from single to multiple* Candida* species isolates, which have been responsible for epidemiological shift in OPC. Infections with* Candida* are almost endogenous; therefore, identification of the species and corresponding susceptibility patterns to antifungal agents can be helpful for the management of these infections. As demonstrated in this study, resistance to azole drugs such as fluconazole, the most frequent antifungal used in the country, could be suggestive of the need for routine fungal culture and* in vitro* drug susceptibility pattern of yeast in medical centers in order to manage more efficiently the invasive candidiasis in the above-mentioned patients.

## Figures and Tables

**Table 1:** Zone diameter interpretation for fungi.

Drug	Potency	Zone diameter in mm		
		Sensitive	Intermediate	Resistant
Clotrimazole	10 *μ*g/disk	≥20	12–19	≤11
Fluconazole	25 *μ*g/disk	≥19	15–18	≤19
Ketoconazole	10 *μ*g/disk	≥28	21–27	≤20
Miconazole	10 *μ*g/disk	≥20	12–19	≤11
Amphotericin B	10 *μ*g/disk	≥15	10–14	≤10
Nystatin 50 *μ*g/disk	50 *μ*g/disk	≥15	10–14	No

**Table 2:** Demographic characteristics of study subjects.

Gender	Male	69 (30.8%)
	Female	155 (69.2%)

Age in years	0–19	43 (19.2%)
	20–29	33 (14.3%)
	30–39	84 (37.5%)
	40–49	47 (21.0%)
	≥50	17 (7.6%)

Under HAART	Male	36 (52.2%)
	Female	76 (49.0%)

**Table 3:** Distribution of yeast isolates in relation to sex.

Species	Female	Male	Total
*C. albicans*	73 (68.9%)	33 (31.1)	106
*C. glabrata *	18 (75.0%)	6 (25.0%)	24
*C. tropicalis *	9 (52.9%)	8 (47.1%)	17
*C. krusei *	2 (50.0%)	2 (50.0%)	4
*C. kefyr *	1 (50.0%)	1 (50.0%)	2
*Rhodotorula* sp.	1 (100)	—	1
*Cryptococcus laurentii *	1 (100)	—	1

Total	105 (67.7%)	50 (33.3%)	155 (100%)

**Table 4:** Distribution of yeast isolates in HIV patients with OPC under HAART and not under HAART (*n* = 155).

Species	Patients under HAART	Patients not under HAART	Under HAART and not under HAART
*C. albicans*	43 (27.7%)	63 (40.6%)	106 (68.4%)
*C. glabrata *	11 (7.1%)	13 (8.4%)	24 (15.5%)
*C. tropicalis *	8 (5.2%)	9 (5.8%)	17 (11.0%)
*C. krusei *	1 (0.65%)	3 (1.9%)	4 (2.6%)
*C. kefyr *	1 (0.65%)	1 (0.65%)	2 (1.3%)
*Rhodotorula* sp.	0 (0.0%)	1 (0.65%)	1 (0.65%)
*C. laurentii *	0 (0.0%)	1 (0.65%)	1 (0.65%)

Total	64 (41.3%)	91 (58.7%)	155 (100%)

**Table 5:** *In vitro* antifungal susceptibility pattern of yeast isolates.

Species	Pattern	Antifungal drugs (*n*, %)					
		Am	CL	Flu	Keto	Mic	NY
*C. albicans *(106)	S	98 (92.4)	105 (99.1)	92 (86.8)	95 (89.6)	101 (95.3)	105 (99.1)
	I	6 (5.7)	0 (0)	7 (6.6)	5 (4.7)	4 (3.8)	1 (0.9)
	R	2 (1.9)	1 (0.9)	7 (6.6)	6 (5.7)	1 (0.9)	0 (0)
*C. glabrata * (24)	S	21 (87.5)	21 (87.5)	19 (79.2)	20 (83.3)	19 (79.2)	22 (91.6)
	I	1 (4.2)	2 (8.3)	1 (4.2)	1 (4.2)	5 (20.8)	1 (4.2)
	R	2 (8.3)	1 (4.2)	4 (16.6)	3 (12.5)	0 (0)	1 (4.2)
*C. tropicalis * (17)	S	9 (53.0)	15 (88.2)	10 (58.8)	13 (76.5)	11 (64.7)	16 (94.1)
	I	5 (29.4)	1 (5.9)	1 (5.9)	1 (5.9)	6 (35.3)	1 (5.9)
	R	3 (17.6)	1 (5.9)	6 (35.3)	3 (17.6)	0 (0)	0 (0)
*C. krusei * (4)	S	0 (0)	3 (75.0)	0 (0)	3 (75.0)	0 (0)	4 (100)
	I	3 (75.0)	0 (0)	2 (50)	1 (25.0)	4 (100)	0 (0)
	R	1 (25.0)	1 (25.0)	2 (50)	0 (0)	0 (0)	0 (0)
*C. kefyr * (2)	S	0 (0)	2 (100)	0 (0)	1 (50)	0 (0)	1 (50)
	I	1 (50)	0 (0)	2 (100)	1 (50)	2 (100)	0 (0)
	R	1 (50)	0 (0)	0 (0)	0 (0)	0 (0)	1 (50)
*C. laurentii*	S	1 (100)	1 (100)	1 (100)	1 (100)	1 (100)	1 (100)
	I	0 (0)	0 (0)	0 (0)	0 (0)	0 (0)	0 (0)
	R	0 (0)	0 (0)	0 (0)	0 (0)	0 (0)	0 (0)
*Rhodotorula* spp.	S	1 (100)	1 (100)	1 (100)	1 (100)	1 (100)	1 (100)
	I	0 (0)	0 (0)	0 (0)	0 (0)	0 (0)	0 (0)
	R	0 (0)	0 (0)	0 (0)	0 (0)	0 (0)	0 (0)

All isolates	S	130 (83.9)	148 (95.5)	123 (79.4)	134 (86.5)	133 (85.8)	150 (96.8)
	I	16 (10.3)	16 (10.3)	13 (8.4)	8 (5.2)	21 (13.5)	3 (1.9)
	R	9 (5.8)	4 (2.6)	19 (12.3)	13 (8.4)	1 (0.6)	2 (1.3)

S: susceptible; I: intermediate; R: resistant; Am: amphotericin B; Clo: clotrimazole; Flu: fluconazole; Keto: ketoconazole; Mic: miconazole; NY: nystatin.
